# Bio-synthesis of bacterial cellulose from ramie textile waste for high-efficiency Cu(II) adsorption

**DOI:** 10.1038/s41598-025-02310-6

**Published:** 2025-05-28

**Authors:** Shihang Ma, Guoguo Xi, Xiangyuan Feng, Qi Yang, Zhenghong Peng, Dong Qiu, Yuqin Hu, Xin Zhao, Lifeng Cheng, Shengwen Duan

**Affiliations:** 1 Institute of Bast Fiber Crops/Center of Southern Economic Crops, Chinese Academy of Agricultural Sciences, Changsha, 410205 China; 2 Beijing National Laboratory for Molecular Sciences, Laboratory of Polymer Physics and Chemistry, CAS Research/Education Center for Excellence in Molecular Sciences, Institute of Chemistry, Chinese Academy of Sciences, Beijing, 100190 China

**Keywords:** Bacterial cellulose, Ramie fibers hydrolysate, Kombucha, Ferment, Copper ions removal, Biological techniques, Biotechnology, Microbiology, Materials science

## Abstract

The current study aims at the high-value utilization of ramie textile waste and explores a bio-synthetic pathway to convert waste ramie fibers into bacterial cellulose (BC). Ramie fibers were treated with commercial cellulase (C2730) and the hydrolysate was used as a base medium (RFH) for BC synthesis by fermentation. The enzymatic hydrolysis parameters were optimized by response surface methodology, yielding an optimal temperature of 40 °C, 64 h, and an enzyme dosage of 5.7%. Under these optimized conditions, the resultant yield of reducing sugars was 31.24 ± 0.37 g/L. And then the *Novacetimonas hansenii* HX1 strain isolated from kombucha was used for fermentation production of BC. The study found that adding yeast extract into RFH can significantly increase BC production, and 7.2 g/L BC can be produced within 7 days. The physical and chemical properties of BC were then analyzed, including Scanning electron microscopy (SEM), Fourier transform infrared spectroscopy (FTIR), X-ray diffraction (XRD) and Thermogravimetric analysis (TGA), confirming its type *I*_α_ cellulose structure and good thermal stability. In particular, BC shows efficient adsorption capacity for Cu(II) ions in aqueous solution, with the highest adsorption efficiency reaching 95.62%. This research not only provides a new way to recycle textile waste, but also lays the foundation for the application of BC in the field of environmental remediation.

**Supplementary Information:**

The online version contains supplementary material available at 10.1038/s41598-025-02310-6.

## Introduction

Biomass utilization is an ecological problem that needs to be solved urgently around the world^1^. One significant type of cellulose-based material is the naturally occurring residual ramie fibers from textile factories. For example, following chemical degumming, between 30 and 40 percent of the abandoned staple fibers are produced in the carding process of ramie fibers, whether the new wool spinning carding process or the traditional spun silk carding process^2^. Moreover, a large amount of waste fabrics will be produced from daily use^3^. If these materials are not fully utilized, it would not only result in waste raw materials but also negatively impact the economic benefits of related enterprises and even the sustainable development of ramie textile industry. Therefore, reusing ramie fibers becomes an urgent issue for the ramie textile industry. It is understood that waste fibers are mainly composed of cellulose, which is polymerized from the structural unit of glucose, and the cellulose content is up to 75%. Previous studies have shown that other agricultural waste biomass can be directionally converted into lactic acid^4^, ethanol^5^, butanol^6^, and other small molecular organic matter^7^; however, until now few studies have been conducted on the microbial fermentation of ramie fibers for biosynthesis.

Bacterial cellulose is an interesting class of microbially synthesized materials^8^, which has been widely exploited in wound dressing, artificial skin, protein delivery, proton exchange membrane, and other functional composites because of its high water retention capacity, good biocompatibility, plasticity, and mechanical stability^9,10^, etc. However, the cost of BC production restricts its popularization and application. Based on the concept of green environmental protection, more and more low-cost materials are applied for the biosynthesis of BC^11,12^, such as pineapple^13^, tobacco^14^, wheat straw^15^, cotton^16^, paper sludge^17^, starch kitchen wastes^18^, natural polymers^19^, mango peel waste^20^, etc. As far as we know, research on the biosynthesis of BC from waste ramie fibers has not been studied previously. Generally, BC is synthesized by the strains from the *Komagataeibacter* genus^21^ as well as non-acetobacter microorganisms, such as *Enterobacter* sp. and *Pseudomonas* sp.^22^, and the Gram-positive bacterium *Lactiplantibacillus plantarum*^23^. Kombucha colony is known to possess a variety of microorganisms that tend to form strong symbiotic relationships and complex metabolic pathways^24^. Therefore, kombucha has significant potential for BC production. Although there have been some studies on the use of kombucha to select excellent strains^25,26^, the yield of BC still cannot meet the needs of industrial production^27^.

With the increasing demand for textile products, the usage of dyes has gradually increased in the field of textiles and dyeing^28^. Nevertheless, synthetic dyes are persistent organic pollutants that can contaminate water bodies, affecting both aquatic life and human health. The presence of these dyes in water can lead to diseases and ecological imbalances^29^. To reduce the risk of such wastewater contamination, a feasible technology with cost-efficiency should be developed to purify wastewater before discharge. Cu(II) is a toxic heavy metal contaminant widely distributed in ecological conditions^30^. Currently, a variety of methods including adsorption, membrane separation, chemical precipitation, and biodegradation have been reported to remove Cu(II) from aqueous solution, among which the adsorption method is considered to be a very effective and inexpensive treatment method, suitable for large-scale industrial applications^31^. Studies have shown that bacterial cellulose, enriched with hydroxyl groups (-OH), possesses a strong adsorption capacity^19,20,32^. By combining bacterial cellulose with bioactive agents sourced from plants or plant residues, the synergistic effect of the different components can further enhance its adsorption performance for heavy metals^33^. However, the preparation of traditional bacterial cellulose often relies on specific strains and more complex processes, and the cost is high, which limits the large-scale application. In this context, it is particularly important to explore new, low-cost and sustainable sources of bacterial cellulose. Ramie waste is rich in polysaccharides such as cellulose, which provides a rich carbon source for bacterial growth. Through a specific microbial fermentation process, it is possible to convert ramie waste into bacterial cellulose. The bacterial cellulose derived from ramie waste not only has the potential capacity of traditional bacterial cellulose to adsorb heavy metals, but also can realize the resource utilization of waste and reduce production costs.

In this work, ramie fibers hydrolysate by cellulase enzymatic action would be utilized as carbon source for BC biosynthesis by the strain isolated from kombucha. The BC-producing strain was identified and its dynamic changes of various indexes in the fermentation process were analyzed. The impact levels of different nitrogen sources on BC yield were studied and then the structural characteristics of BC and its potential as a biosorbent to remove heavy metal Cu(II) ions from aqueous solution with different pH values were discussed. Briefly, it is expected to provide technical support and theoretical guidance for BC fermentation process optimization, industrial production and further application as well as the high value utilization of textile wastes.

## Materials and methods

### Raw materials and reagents

All ramie fibers were collected from the modern agricultural technology experimental demonstration base in Yuanjiang City, Hunan Province and stored in the laboratory. Commercial cellulase (C2730) and DNA extraction kits were purchased from Sigma-Aldrich and Novozymes (China) Biotechnology Co., Ltd. and Sangong Biotech (Shanghai) Co., Ltd., respectively. Unless otherwise stated, other reagents were obtained from Beijing Solebaum Technology Co., Ltd., such as Agar, yeast, peptone, disodium hydrogen phosphate, citric acid, glucose, etc. Copper nitrate trihydrate was purchased from Macklin Inc. (Shanghai, China).

### Isolation and identification of strain

#### Isolation

With reference to the previous literature^25^, BC-producing strain was isolated from kombucha at 30 °C for 3 days by plate screening on solid medium after enrichment in traditional Hestrin-Schramm (HS) medium (g/L)^34^: glucose (20.0), yeast extract (5.0), peptone (5.0), disodium hydrogen phosphate (2.5), agar (1.5) and citric acid monohydrate (1.15) at pH 5, 30 ± 1 °C for 48 h. After obvious bacterial colonies grew in the plate culture medium, one colony was selected and placed into the liquid medium for incubation, and the culture medium containing gel membrane was selected to repeat the above steps until the colonies in the plate were uniform in shape and size and had stable ability to produce gel membrane. After being cultivated for 3 days, the single bacterial colony was transferred on an agar slant, and the fermentation liquid was kept in glycerol before being stored in the refrigerator.

#### Physiological and biochemical characterization

After activating the isolated strain stored on the inclined plane, a single colony was prepared by coating or plate scribing method in a super-clean table. After incubating at 30 °C for 3 days, the colony morphology was observed. The activated single colony was selected with the inoculation ring, evenly mixed in the normal saline on the slide, and baked on the alcohol lamp. The bacteria were stained by Gram technique and observed under the microscope. The physiological and biochemical characteristics were detected according to the reported method^35^, including contact enzyme test, oxidase test, acetic acid oxidation test, ethanol oxidation test, exercise test, lactic acid oxidation test.

#### Molecular identification

The selected strain was frozen in glycerol, thawed and shaken, and 0.2 mL of the storage solution was absorbed into HS liquid medium at 30 °C and activated for culture at 150 rpm. The culture was tested at 600 nm absorbance in a ELISA PLATE, and the culture with an OD_600_ value of 0.6 was obtained by centrifugation. The template DNA of the selected strain was obtained by boiling with 1 mL sterile water for 5 min and freezing at − 20 °C for 5 min. Then, the 16S rDNA sequences were amplified with bacterial universal primers 27F (5′-AGA GTT TGA TCC TGG CTC AG-3′) and 1492R (5′-GGT TAC CTT GTT ACG ACT T-3′). The polymerase chain reaction (PCR) amplification system (25 μL) included 12.5 μL of 2 × Es Taq MasterMix reaction solution, 1 μL of each primer, 5.5 μL of double distilled H_2_O, and 5 μL of template DNA. The PCR amplification was followed by initially denaturating at 94 °C for 2 min, denaturation at 94 °C for 30 s, annealing at 55.4 °C for 30 s, extension at 72 °C for 30 s, and a final extension of 72 °C for 2 min. The three reactions of denaturation, annealing, and extension went through 35 cycles. The phylogenetic tree of 16S rDNA gene was established using MEGA 7.0 software and the neighbor-Joining method was used to identify the strain species.

#### Assessment of strain growth and BC yield

The strain frozen in glycerol was activated in HS medium and then diluted in gradient. Afterwards, one colony was selected to be inoculated in HS for 24 h, and inoculated sequentially at 5% in a conical flask containing 50 mL medium. They were cultured in an incubator at 30 °C, and the absorbance was measured by time-course sampling method on a spectrophotometer at the wavelength of 600 nm and the growth curve of strain was drawn according to the analysis of absorbance value. The productivity of reducing sugar was determined by DNS (3,5-dinitrosalicylic acid) method^36^. Add 2 mL of the diluent to be tested and 1.5 mL of the DNS reagent into a 25 mL volumetric bottle and heat it accurately in a boiling water bath for 5 min. Immediately after the reaction is completed, it is quickly cooled to room temperature with ice water bath, and then filled with deionized water to the scale line, thoroughly mixed and transferred to the colorimetric dish. The absorbance of the mixed solution was determined by spectrophotometer at 540 nm wavelength to quantitatively analyze the reducing sugar content^37^. The BC film was washed, placed in 0.2 M NaOH, and heated in an 80 °C water bath for 30 min to remove residual medium and bacterial residue, and then repeatedly washed or soaked with deionized water until the neutral pH. The BC film is then dried in a freeze-dryer. The BC dry weight yield is then weighed and calculated (i.e., the dry matter weight of 1 L of liquid medium producing BC, in g/L).

### Utilization of ramie fibers hydrolysate for bacterial cellulose production

#### Optimization of enzymatic hydrolysis condition

Inspired by enzymatic saccharification of biomass, cellulase was used to hydrolyze ramie fiber^38–40^. Ramie fibers were cut into 2–3 cm lengths and was further hydrolyzed to release monomeric sugars. Specifically, the hydrolysis experiments were carried out by using 5% (*w*:*v*) fibers loading and 3, 4, 5, 6, 7% (*v*:*v*) commercial cellulase concentration and a shaker agitation rate of 150 rpm. The pH was adjusted to 5 using a mixture of disodium hydrogen phosphate and citric acid monohydrate. The hydrolysate was then separated insoluble solids from fibers hydrolysate by using filtration and centrifugation for subsequent base medium. The effects of enzymatic conditions (e.g., temperatures, times, and enzyme concentration) on the fibers hydrolysis efficiency were explored. Based on single factor experiment, three factors, namely, temperature, time, and enzyme activity, which had obvious effects on reducing sugar content were selected as independent variables, and reducing sugar content in the hydrolysate served as the response value, and then the three-factor, three-level Box-Behnken experimental design were performed by the Design-Expert 13 software. The detailed information on experimental design of response surface methodology (RSM) is provided (see “Supplementary Material”).

#### Bacterial cellulose production in ramie hydrolysate medium

The ramie fibers hydrolysate without other chemical components was used as the ramie hydrolysate base medium, which was marked as RFH medium. The selected strain was cultured at 30 °C and 150 r min^−1^ for 24 h in a total volume of 50 mL HS medium. Then, the activated seed solution was added to 50 mL ramie hydrolysate medium with an inoculum content of 5% and then subjected to static fermentation at 30 °C. Bacterial concentration, reducing sugar content, and BC yield were determined and recorded at 48 h intervals.

#### Screening of nitrogen sources to enhance BC yield

Studies indicate that the nitrogen source utilized is a key factor influencing the production of BC besides carbon source^41,42^. In order to study the effect of different nitrogen sources on yield, ramie hydrolysate based medium was supplemented with 5 g/L urea, extract of yeast and beef, peptone, corn starch and soybean as nitrogen sources, respectively. BC fermentation method was stated in the section “Bacterial cellulose production in ramie hydrolysate medium”. The freeze-dried BC membrane was chosen as the index to evaluate the effectiveness of nitrogen sources. The optimal added nitrogen source for RFH medium was determined according to the highest BC yield.

#### Characterization of bacterial cellulose

Micromorphology was observed using cold field-emission scanning electron microscopy (SEM, JSM-7500F, JEOL) with an energy-dispersive X-ray spectroscopy (EDS) operated at 3 kV. Fourier transform infrared spectra (FTIR; IRSpirit, Shimadzu) was examined via Cary 600 Series (Agilent Technologies, Santa Clara, USA) with an IR mode of 4 cm^−1^ resolution. Constituent phases and crystallinity were analyzed through X-ray diffraction (XRD, SmartLab SE, Rigaku) using Cu K*α* radiation (λ = 1.5418 Å) operated with the scanning rate of 2° min^−1^ at 40 kV and 200 mA. The interplanar distances (*d*) and grain size can be obtained through the Bragg’s law Eq. (1) and Scherrers formula Eq. (2), respectively.

1 $$d=\frac{\lambda }{2{\sin}\theta }$$

2 $$D=\frac{0.9\uplambda }{\text{FWHM}\times \text{cos}\theta }$$

where *θ* is the diffracted angle (°), and *λ* is the X-rays wavelength (nm), FWHM is the peak width at half the maximum height (°), *D* is the crystallite size corresponding to the diffracted plane (nm). Crystallinity was calculated by the method described in previously reported work^43^. Crystal allomorphs were determined according to *Z* discriminant function (3)^25^.

3 $${\rm Z}=1693 {d}_{1}-902 {d}_{2}-549$$

where, *d*_*1*_ is the spacing of crystallographic plane (100), and *d*_*2*_ is the spacing of crystallographic plane (010). TA SDT 2960 (TA Instruments) was employed to record the thermogravimetric (TG) curves in open α-alumina pans at N_2_ atmosphere with a flow rate of 70 mL min^−1^ at a heating rate of 10 °C min^−1^. Jade-DSC (Perkin Elmer) equipped with intercooler system 2P was used to perform differential scanning calorimetry (DSC) analysis with a heating rate of 10 °C min^−1^ and nitrogen flow rate of 50 mL min^−1^. The zeta potential of BC samples was determined by Malvern Zetasizer (BeNano, Shimadzu).

#### Removal of Cu(II) from aqueous solutions

The hydroxyl groups (-OH) on the surface of bacterial cellulose bestow upon it a strong adsorption capacity^18^. Herein, heavy metal Cu(II) ions were used to determine the adsorb capacity of BC. The Cu(II) standard solution of 1000 mg/L was purchased from the Standard Material Center of the Chinese Academy of Metrology and diluted to 10 mg/L with deionized water for the subsequent experiment. A mixtures of 1 mg of freeze-dried adsorbent and 100 mL of heavy metal solution in a 250 mL conical flask were oscillated using thermostatic shaker (MaxQ4000, Thermo scientific, America) at 25 °C and 100 rpm for 24 h. The adsorption experiments was performed using an inductively coupled plasma optical emission spectrometer (ICP-OES; ICPE-9820, Shimadzu) at 324.754 nm as *λ*_max_ of Cu. The metal adsorption amount by BC (*Q*) from aqueous solution at any time (*t*) was calculated as the following Eq. (4):

4 $${Q}_{t}=\frac{V\left({C}_{0}-{C}_{t}\right)}{m}$$

where *C*_0_ is the initial concentration (mg/L), *C*_t_ correspond to the concentration after absorbent time *t* (mg/L), *V* is the volume of aqueous solution (L) and *m* is the mass of freeze-dried BC (g).

### Data statistical analysis

The software of SPSS Statistics 25, Origin 2021 and Analysis of Variance (ANOVA) were used for data processing and significance analysis (*p* < 0.05). Design-Expert 13 was used for data processing and analysis in response surface experiments.

## Results and discussion

**Fig. 1:**
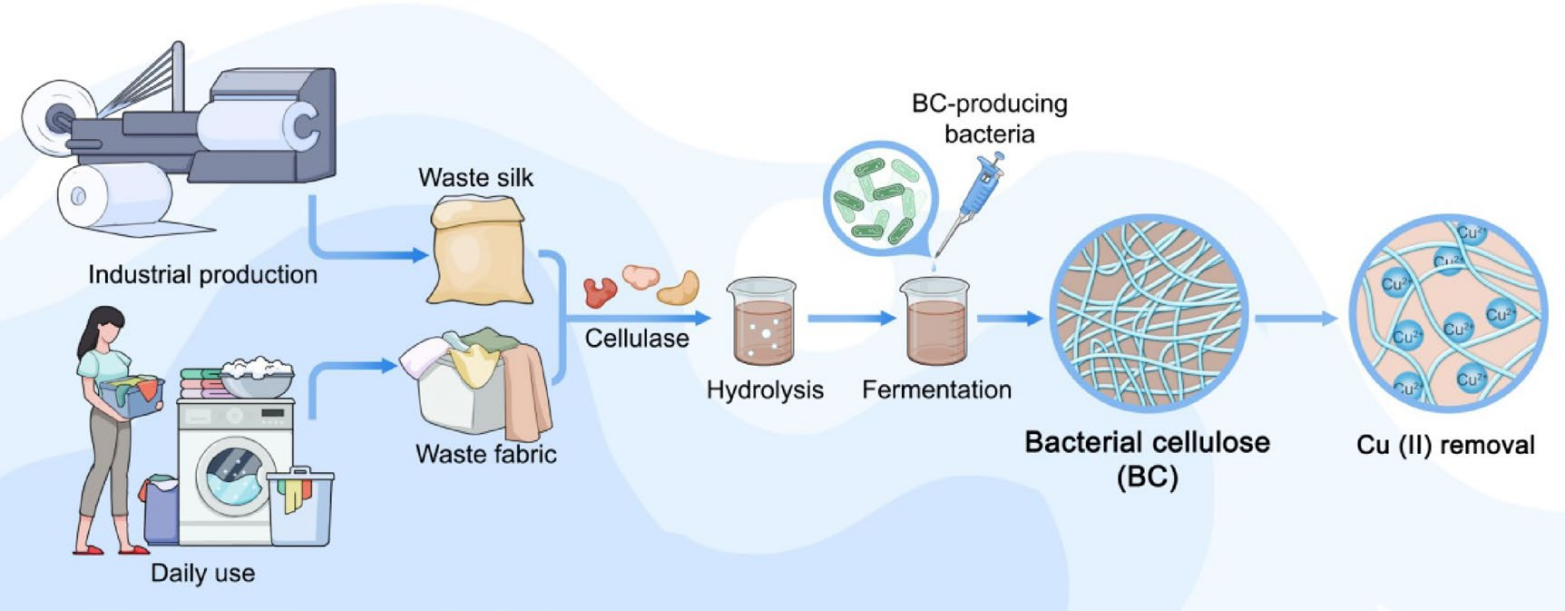
A simple plane for the production of BC from waste ramie fibers and fabrics to minimize the copper ions pollution from textile dyeing.

In the present study, a simple, clean, and green process was developed to produce BC from waste textile fibers and ramie fabrics for minimizing the heavy metal Cu(II) pollution from textile dyeing chain. Ramie bast contains a large number of impurities, which are mainly polysaccharides gum substances. The hemicellulose, pectin, lignin and water soluble content of raw ramie were reported to be 14.69% ± 0.98%, 4.86% ± 0.69%, 2.08% ± 0.57%, and 5.96% ± 0.36%, respectively. These gums in the bast need to be removed before spinning, so that the ramie fibers separate from each other to form a fiber. However, from the point of view of releasing reducing sugar through cellulose hydrolysis by cellulase, the degumming or not of degumming fiber has no great influence on the yield of reducing sugar. Therefore, the ramie planted in farmland was selected as the object of this study after drying and intervention treatment. The procedure roadmap is illustrated in Fig. 1. The process involves enzymatic hydrolysis of ramie fibers and fermentation of bacteria cellulose (BC) and exploration of its physical adsorption scenarios.

### Strain classification

**Fig. 2:**
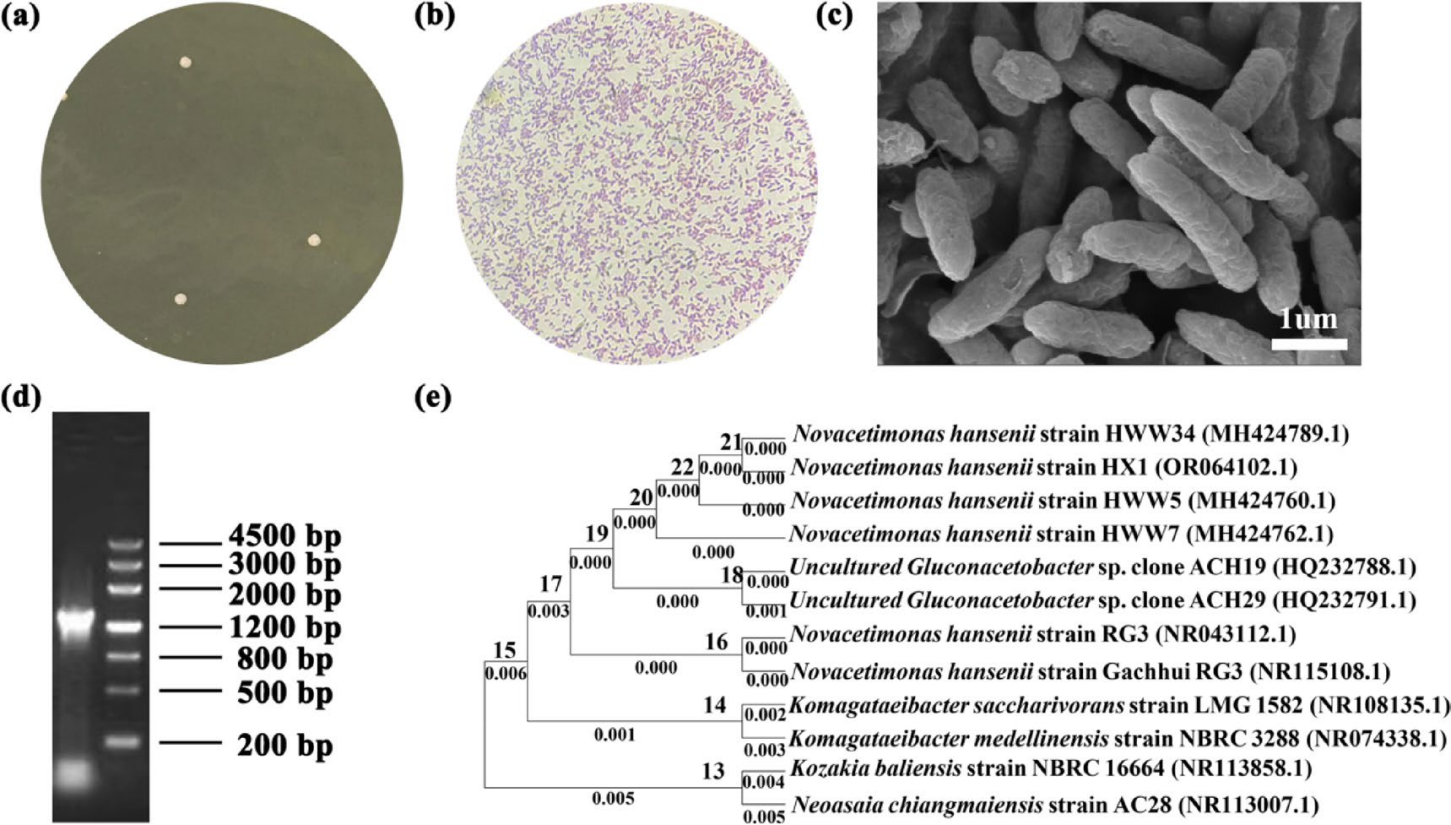
(**a**) Colony morphology. (**b**) Gram-staining character. (**c**) Scanning electron microscope image. (**d**) Electropherogram pattern. (**e**) Phylogenetic tree based on 16S rDNA gene sequences of PCR amplification products.

We preliminarily isolated a nanocellulose-producing strain from kombucha strain and further conducted identification experiments. As shown in Fig. 2a, the colony of unknown bacteria isolated from kombucha showed a regular round shape with a raised surface and an opaque milky white color when growing on the fermented solid medium. The Gram-staining result showed negative (Fig. 2b). Subsequent morphological observation revealed that the unknown bacterium had a rough surface and was approximately rod-shaped (Fig. 2c), which was consistent with the morphological characteristics of *Komagataeibacter* isolated from kombucha for BC production^44^. The experimental results of physiological identification of the Gram-negative strain are shown in Table S1. The isolated strain demonstrated catalase-positive and oxidase negative, which was similar to previously reported cellulose-producing bacteria isolated from kombucha strain^25^. A milky calcium carbonate halo was produced around the colony both acetic acid and lactic acid oxidation tests (Fig. S1a, b), which makes it easier to identify the isolated strain as the family *Acetobacteraceae*, since *Acetobacter* could oxidize lactate to form carbonate^45^. This result seems to be inconsistent with that of morphological feature analysis and assay results of contact enzyme and oxidase, which can be well explained by published work that *Komagataeibacter* is one of the most bio-relevant genera in the *Acetobacteraceae* family^46^. Moreover, the isolated bacteria can convert ethanol to acetic acid by peroxidation in neutral reaction (bromophenol blue indicator turns yellow, which was shown in Fig. S1c). After puncture inoculation, the bacteria could only grow on the surface of the medium and could not survive through the medium, indicating that the bacterium was not motility. Further molecular identification of the isolated strains was performed. Electrophoretic pattern of the DNA extracted from isolated strain shows a neat band under ultraviolet conditions and the length of the PCR-amplified fragment is about 1350-bp (Fig. 2d). The original full-length imprinted image is placed in Fig. S2 of the supplementary information file. Based on the analysis of 16S rDNA, this strain had 99% sequence identity with the 16S rDNA sequences of *Novacetimonas hansenii* HWW7, *Novacetimonas hansenii* HWW34 and *Novacetimonas hansenii* HWW5 (Fig. 2e). Brando et al. found that several species of *Komagataeibacter* maybe belong to the genus *Novacetimonas,* which was supported by phylogenomic and comparative analyses^47^. Therefore, it can be reasonably inferred that the molecular identification results are not inconsistent with the above morphology and physiological and biochemical analysis. Given the above, the isolated strain was preliminarily named as *Novacetimonas hansenii* HX1. The number of NCBI accessions is OR064102.1.

### Growth dynamic analysis of *Novacetimonas hansenii* HX1 strain

**Fig. 3:**
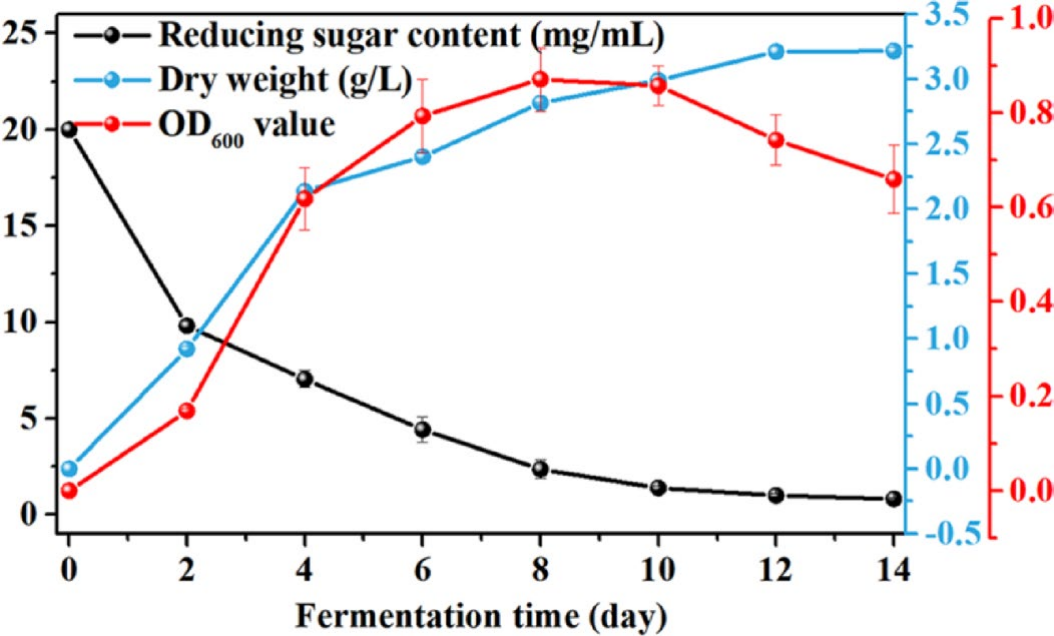
Variations in the dried weight of bacterial cellulose, bacterial concentration, and reducing sugar during the fermentation process of *Novacetimonas hansenii* HX1.

Bacterial growth curves are essential to describe bacterial growth and metabolism behavior. We investigated the growth kinetics of *Novacetimonas hansenii* HX1 in HS medium under the static culture condition of 30 °C. Figure 3 shows the cell growth curve of *Novacetimonas hansenii* HX1 was *S*-shaped. The bacteria were in the logarithmic growth phase within 2–4 days and then reached the decline phase after 10 days. When fermented, those bacteria in logarithmic period were selected as strain seeds, which could not only shorten the lag period of the fermentation process but also facilitate higher bacterial activity. Thus, the bacterium with a species age of 4 days and OD value of about 0.6 was selected as seeds in the subsequent fermentation for BC production. However, this strain currently faces a major challenge of the long fermentation cycle. Our group would further explore the culture conditions and the growth mechanism of HX1, and explore more efficient media to shorten the strain growth period and meanwhile increase the BC yield. Note that, the experimental results also show the change curve of various fermentation parameters. When cultured within 8 days, the variation trend of OD value was similar to the yield of BC, confirming the synchronization nature of biosynthesis of BC and bacterial growth. As shown in Fig. 3, the sugar consumption curve was reversed S-shaped. Considering that glucose is commonly utilized as a nutrient source for bacterial growth and biosynthesis of BC, it might be inferred that the total soluble sugar in the medium is the limiting substrate for the growth of *Novacetimonas hansenii* HX1, and the synthesis of BC belongs to the growth-coupled type, that is, type I fermentation proposed by Garden^48^. The results would guide the process optimization of BC biosynthesis and further industrial production.

### Enzymatic hydrolysis of ramie fibers

**Fig. 4:**
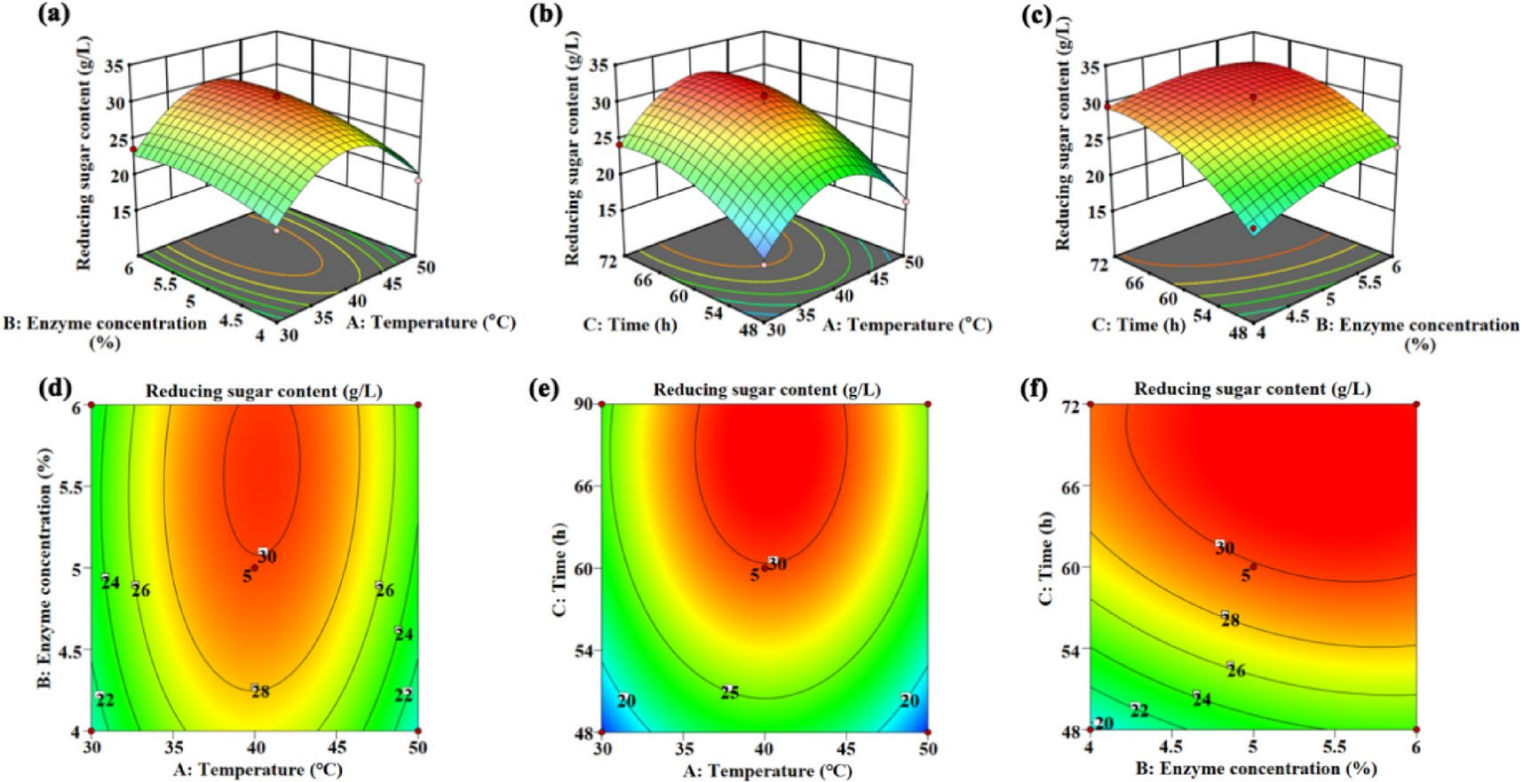
(**a**) Response surface for temperature vs. enzyme concentration, (**b**) Response surface for temperature vs. time, (**c**) Response surface for enzyme concentration vs. time. (**d**) Contour map for temperature vs. enzyme concentration, (**e**) Contour map for temperature vs. time, (**f**) Contour map for enzyme concentration vs. time.

Enzymatic hydrolysis is an efficient and environmentally friendly method for degrading natural fibers biomass to produce reducing sugar, which is a key component of BC production medium. As known, cellulase is a complex enzyme that could degrade cellulose^49^. Thus, it is beneficial to improve the utilization rate of ramie biomass by exploring the optimum hydrolysis conditions of cellulase for ramie fibers. In this experiment, hydrolysis temperature, the enzyme concentration and reaction time were used as the indexes to produce reducing sugar, and the best hydrolysis process of ramie fiber by cellulase was studied. Initially, a single factor experiment was conducted to analyze the effect of the above three parameters on the yield of reducing sugar (Fig. S3). The result and the corresponding discussion section are placed in the Supplementary Material. On account of cost–benefit analysis, 40 °C, 60 h, and 5% enzyme addition were selected as better optimum values for subsequent tests. The relationship between preparation variables and the yield of reducing sugar was comprehensively evaluated by regression coefficient and square error analysis as well as multi-variable interactions designed by Box-Behnken (described in Tables S2–S4 of the Supplementary Material). Three-dimensional response surface plots are the graphical representation of the regression equation, which could describe the relationship among test parameters and the interaction between response values and the variables. As shown in Fig. 4, the optimal hydrolysis conditions predicted by the response surface method were as follows: temperature 40.4 °C, time 63.8 h, the enzyme concentration of 5.7%, the reducing sugar content in the hydrolysate reached about 31.18 g/L. The experimental results showed that adding a concentration of cellulase of 5.7% made the yield of reducing sugar reach the maximum of 31.24 ± 0.37 g/L at 40 °C after 64 h. The verified experimental results agree well with the calculated values of the established regression equation, which suggests that the equation could be employed to predict and optimize the enzymatic hydrolysis of ramie fiber. The yield of bacterial cellulose are superior to other reported strategies from biomass/industrial-waste enzymatic saccharification, such as lotus roots^50^, molasses^51^ and a mixture of apple waste and tea^52^. The cost of bacterial cellulose production mainly depends on the carbon source, by contrast, the price of biomass and industrial waste is negligible. Thus, the cost for bacterial cellulose production could be significantly reduced by using ramie fibers.

### Optimization of ramie fibers hydrolysate medium

**Fig. 5:**
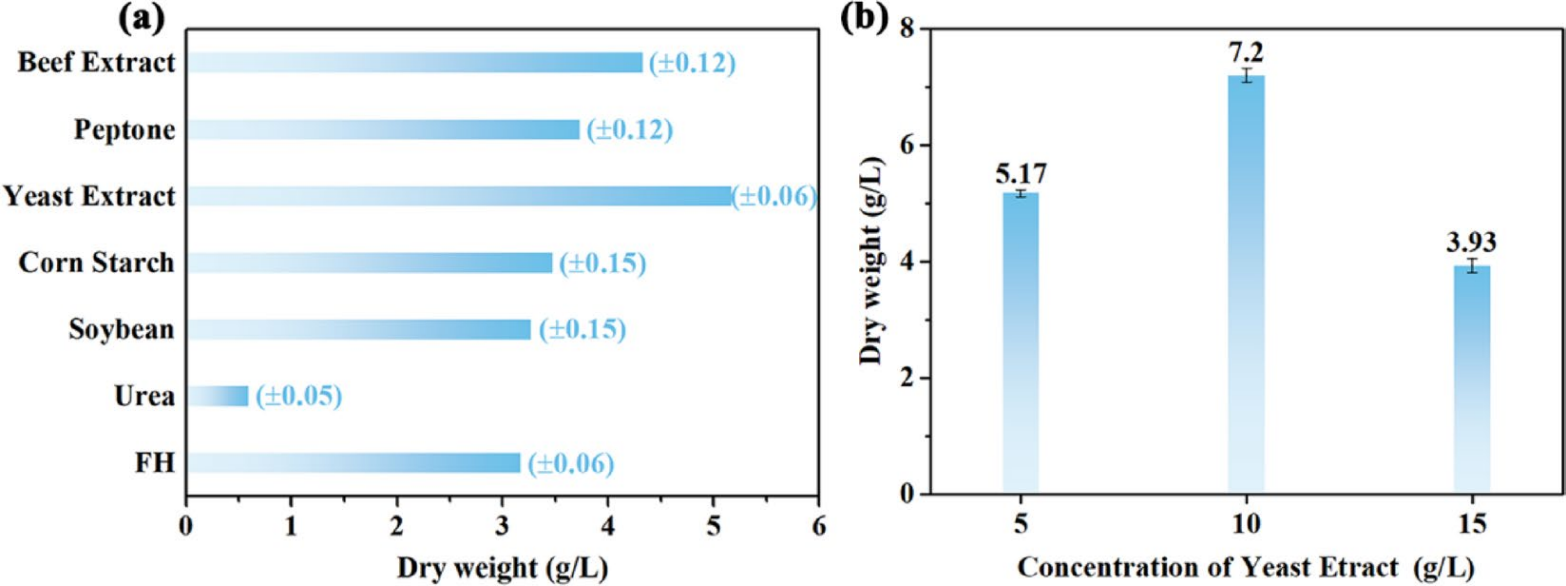
(**a**) Effects of different nitrogen sources (5 g/L) in RFH medium on BC dry weight of HX1 strain after 7 days culture. (**b**) Effect of yeast extract content on BC yield.

As shown in Table S5, the cultivation time for harvesting 3.2 g/L BC by *Novacetimonas hansenii* HX1 strain was shortened from 14 days for HS to 7 days for RFH. Given that RFH can merely be used as carbon source rather than nitrogen source, this may limit BC production to some extent. To further improve productivity, additional nitrogen sources need to be supplemented on the biosynthesis process in RFH medium. As shown in Fig. 5a, the supplementation of RFH with diverse organic nitrogen sources results in the enhancement of BC yield. Among them, the BC yield from RFH containing increased significantly to 5.17 g/L, achieving the best BC production capacity. However, the mass of BC produced in RFH with urea is less than that of the medium without inorganic nitrogen source, suggesting that urea plays inhibitory rather than participation in synthesizing process of BC, thus it may not be appropriate to add inorganic nitrogen sources for BC production, which was verified by previous report^53^. Notably, the productivity of BC was up to 7.2 g/L along with adding 10 g/L of yeast extract within RFH (Fig. 5b), which greatly improved the production efficiency of BC.

Supplementation of RFH with 10 g/L of yeast results as nitrogen source in the enhancement of BC yield on 7-day-continuousday culture (up to 121.88%, i.e. 7.2 g/L). No other chemical components were added despite the yeast extract supplementation, while the HS medium is formulated with glucose, yeast extract, peptone, disodium hydrogen phosphate and citric acid monohydrate. Above results contribute to discussing the enormous potential of using textile-industry byproducts and waste fabric in BC production. In the case of low-cost waste, numerous materials are applied to prepare extracts to achieve satisfactory BC concentrations. For example, Pacheco et al. used HS medium supplemented with cashew residues obtaining 6.0 g/L BC in 7 days^54^. Saleh et al. found starch kitchen waste hydrolysate as a low-cost effective medium for 0.3 g/L/day of BC production^17^. Machado et al. produced 4.0 g/L BC by *K. rhaeticus* in 5 days by partially replacing glucose with sugarcane molasse in the HS medium^55^. Using the bacteria isolated kombucha strain, Leonarski et al. obtained the BC production of 2.3 g/L after 12 days in an alternative medium using extract obtained from acerola waste (5% *w*/*v*)^25^. In short, the possibility of utilizing textile residues is a promising alternative for reducing BC production costs.

### Characterization of bacterial cellulose

**Fig. 6:**
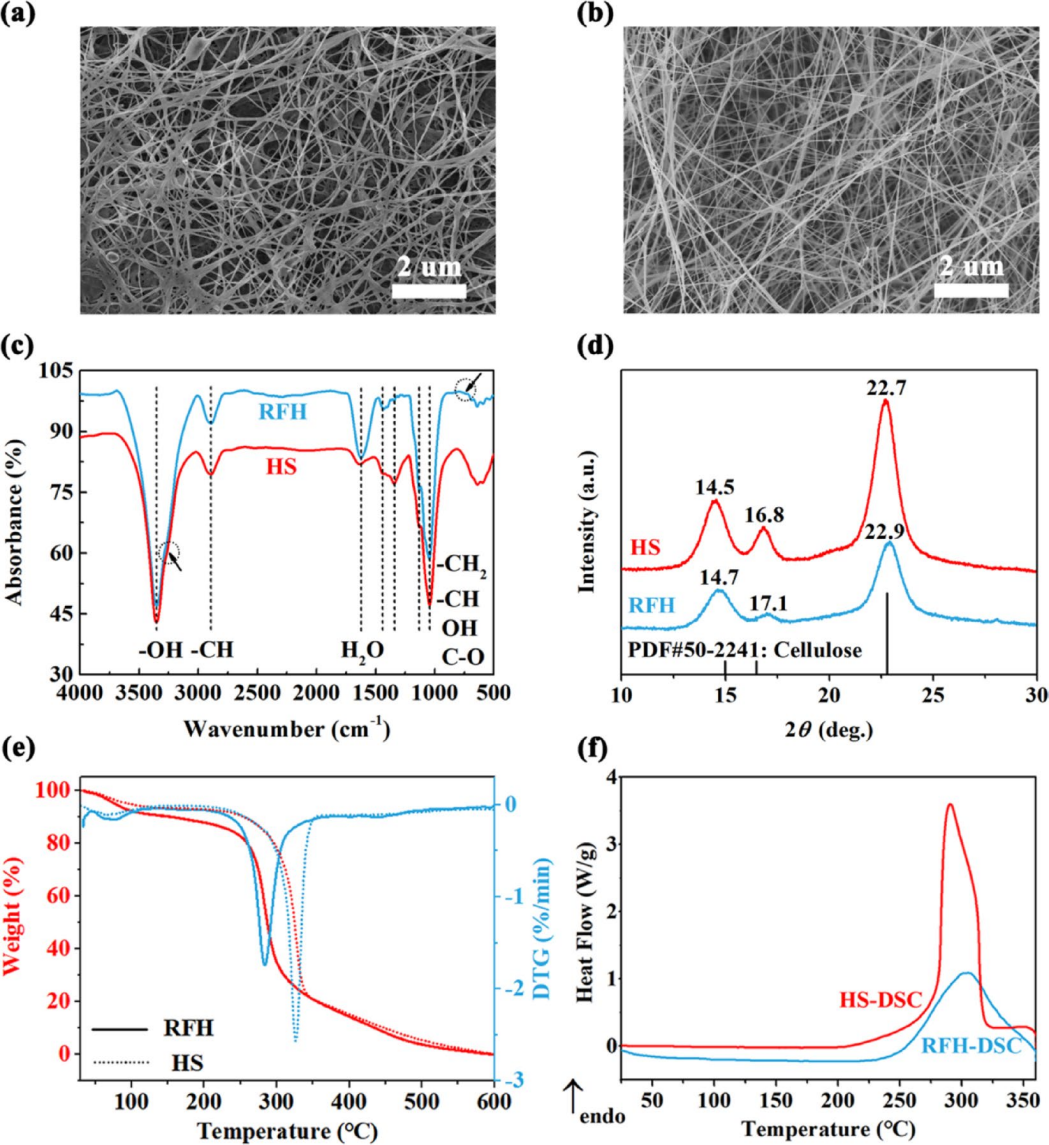
(**a**) SEM image of BC from RFH. (**b**) SEM image of BC from HS media. (**c**) FTIR patterns, (**d**) XRD patterns, (**f**) DSC curves of BC from RFH (as denoted by blue line) and HS (as denoted by red line) media. (**e**) TGA curves of BC produced in RFH (as denoted by solid line) and HS (as denoted by dotted line) media.

Under static conditions, *Novacetimonas hansenii* HX1 strains formed pellicles in the optimized RFH medium (Fig. S4a,b). The SEM images of the obtained freeze-dried BC samples are shown in Fig. 6a,b. The overall morphological structure was influenced by the cultivation medium used. The use of various media leads to significant changes in fiber diameters. The individual fiber diameters of samples for HS and RFH, as seen in Fig. S5a,b, are 42.99 ± 1.35 nm and 54.98 ± 1.37 nm, respectively. Moreover, the fibre became less densification when BC pellicles were produced in RFH media. Notably, the RFH-derived BC displayed very porous pellicles.

FTIR spectroscopy results (Fig. 6c) showed both freeze-dried samples derived from different media own characteristic peaks of cellulose^56^. The obvious peaks were observed at 3350 cm^−1^ (O–H stretching), 2892 cm^−1^ (C–H stretching), 1621 cm^−1^ (H–O–H bending vibration of absorbed water molecules) and 1042 cm^−1^ (C–O stretching)^25^. The band found at 1436, 1339 and 1131 cm^−1^ could be ascribed to symmetric (–CH_2_) bending vibration, O–H in-plane bending and cellulose C–O–C bridges, respectively^43^. Weak peaks centered on 3220 cm^−1^ and 750 cm^−1^ are thought to be responsible for the triclinic *I*_α_ allomorph^43^.

Similar structural features have been observed in previous studies using different media, indicating that the chemical structure is compatible with BC, indicating that the RFH-derived sample is compatible with BC^57^. As shown in Fig. 6d, XRD patterns revealed that both samples exhibited typical diffraction peaks corresponding to the (100), (010) and (110) crystallographic planes of type-I cellulose located at 2*θ* = 14.8°, 17.0°, and 22.8°, respectively^58,59^. Since the peak strength 2*θ* at 14.7° is higher than that at 17.0°, it is easier to identify the obtained cellulose with *I*_α_ type structure in both samples^60^. Furthermore, the crystal structure parameters of produced BC samples were calculated from the resulting XRD peaks and have been included in Table S5. The crystallinity of BC produced from RFH and HS was calculated to be 68.05% and 82.09%, respectively. The lower crystallinity means that BC has good plasticity, which could expand its application potential in new biomedical materials such as wound dressings^61^. The *I*_α_-rich BC sample from RFH can also be verified by the calculated Z value (+ 31.65, listed in Table S6), which is a parameter to distinguish whether BC is enriched in *I*_α_ or *I*_β_ type^62,63^. Besides, both the crystallite size of the peaks near 17° of the two BC samples showed the largest and the numerical difference is not significant. Nevertheless, the average grain size (*D*) was 5.6 nm for RFH and 7.7 nm for HS, indicating a smaller crystallite size of RFH-derived BC. The fitted *D* value from XRD patterns further confirmed that the BC produced in RFH medium has good plasticity^64^, which is consistent with *Z*-value discrimination.

TGA results (Fig. 6e) revealed that both samples own typical thermal degradation behavior of BC^43^. A weak mass loss taking place from room temperature to 120 °C was attributed to the dehydration of physically adsorbed and/or hydrogen bond linked water molecules^25,65^, while a significant mass loss investigated from 200 to 400 °C was ascribed to thermal decomposition of cellulose^66^. In fact, BC samples are stable until temperature increased up to 250 °C, and then start to decompose beyond this temperature. An endothermic peak was observed at 283 °C for RFH and 326 °C for HS as well as a very slower value for the hydrolyzed ramie BC. Seems that residual ramie fibers or other compounds not mentioned in the source raw material still trapped in the cellulose structure, eventually residues from hydrolytic process. Generally, the extrapolated onset temperature (*T*_onset_) and the temperature of maximum weight loss rate (*T*_max_) are accepted as the criteria for thermal stability. As shown in Table S7, RFH-derived BC showed lower values of *T*_onset_ (268 °C) and *T*_max_ (283 °C) than those of HS, indicating its slightly lower thermal stability. This observed result may be due to the low crystallinity of RFH-derived BC obtained from the above XRD analysis^43^. This thermal behavior characteristic of HS-derived sample was also confirmed by the following DSC analysis. Notably, about 20% of BC was left undecomposed up to 350 °C for both samples, indicating a slightly lower purity of sample from both mediums. Seems that the compounds originating from residual ramie fibers and the medium feedstock source still trapped in the cellulose structure, eventually residues from hydrolytic process.

DSC measurements were conducted to estimate the absorption and emission of heat when materials undergo a thermal reaction. The thermal behaviours of glass transition and water molecule loss cannot be identified from the DCS curves of both samples^25^. As shown in Fig. 6f, only an endothermic peak associated with the melting point (*T*_m_) was observed at 305 °C for RFH and 291 °C for HS, which is consistent with the results of native BC^67^. Besides, it can be observed a higher melting enthalpy (∆*H*) of BC from RFH (81.34 J/g) than HS (101.86 J/g), indicating the lower heat resistance of RFH-derived sample^68^.

### Removal of Cu(II) from aqueous solution

**Fig. 7:**
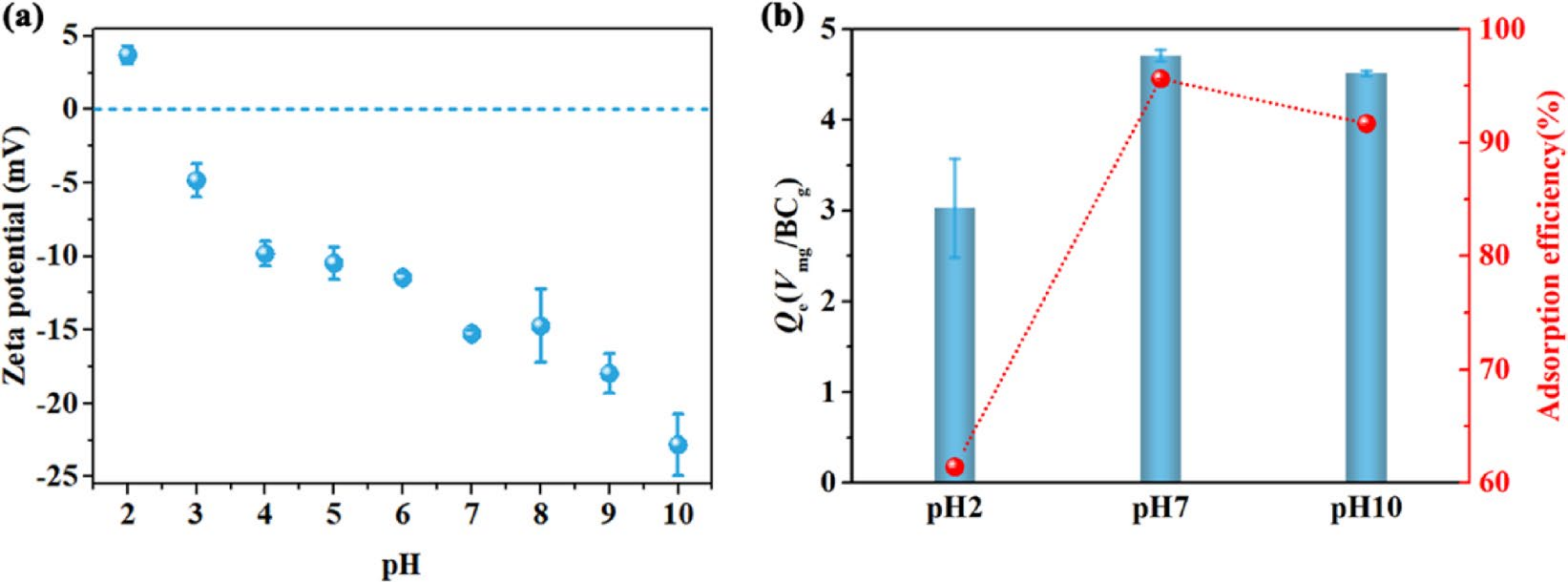
(**a**) The Zeta potential of BC produced by RFH in the pH range of 2–10. (**b**) Adsorption analysis of Cu(II) by BC at different acidity and basicity.

Printing and dyeing processes generate effluent from textile industry containing heavy metals pollution (such as copper, chromium, lead, antimony, arsenic, etc.), which mainly comes from metal-complex dyes and chemical auxiliaries^69^. In view of this, we further evaluated the adsorption potential of RFH-derived BC for cleaning the heavy metal ions (Cu(II)) from aqueous phase. Studies have shown that pH affects the adsorption process mainly by changing the surface charge and the ionization degree of the surface functional groups of the adsorbent^31^. Thus, it is necessary to understand the variation trend of charge value for BC samples as a function of pH. The zeta potential of sample was analyzed at pH 2–10. As shown in Fig. 7a, the point of zero charge (pH_PZC_) of RFH-derived BC was observed to be 3.7, which is close to the previous report^13^. We also investigated the influence of acidity and basicity on the adsorption of Cu(II) on RFH-derived BC. As the pH increases, a rise in the adsorb-capacity of Cu(II) is observed from a low clearance rate of 61.42% at pH 2.0 up to 95.62% at pH 7.0 (Fig. 7b). This could be interpreted by the unique structure and surface characteristics of BC, that is, on the one hand, RFH-derived BC possesses a high specific surface area, which would enrich the physical adsorption sites of adsorption reactions^69^; on the other hand, the negative charge on the adsorbent surface increases with pH rising, which may be advantageous for electrostatic attraction of positively charged phases such as Cu^2+^ species and Cu(OH)_2_. It should be noted that the high uptake rate of Cu(II) at pH value greater than 7 is mainly due to the precipitation of Cu(OH)_2_ rather than the adsorb-capacity of RFH-derived BC^31^. The SEM images and EDX pattern of BC after (Fig. S6a–c) and before (Fig. S6a-c and S6d-f) adsorption of Cu(II) were also investigated. The SEM images clearly illustrate the morphological changes of BC before and after Cu(II) adsorption. The EDS pattern further confirms the presence of Cu on the BC surface after adsorption, indicating the successful Cu(II) adsorption by BC. It is also evident from morphology images that BC did not change significantly after Cu(II) adsorption. The observed morphological stability of BC post-adsorption suggests its potential for reusable applications without significant structural alteration. Moreover, the EDS analysis, coupled with the SEM images, strengthens the argument for the effectiveness of BC as an adsorbent for Cu(II). The retention of BC structure after adsorption underscores its robustness and suitability for repeated use in similar adsorption processes, which is a crucial aspect for practical and economic viability in environmental remediation efforts. The data presented thus contribute to a better understanding of BC’s adsorptive properties and its potential applications in metal ion removal from aqueous solutions. To our knowledge, BC derived from ramie textile waste has adsorption properties comparable to those of eco-friendly cellulose derivatives^70^. Therefore, RFH-derived BC could be a potential substitution for Cu(II) cleaning in textile industry effluent.

## Conclusion

This study successfully biosynthesized bacterial cellulose (BC) from waste ramie fiber through hydrolysis of cellulase using the strain *Novacetimonas hansenii* HX1 selected in kombucha. The ramie hydrolysis conditions were optimized through response surface methodology, and the production efficiency of BC was significantly improved by using the hydrolyzate and the fermentation cycle was shortened. In addition, we also explored the effects of different nitrogen sources on BC production and found that the addition of yeast extract significantly increased BC production. Through physical and chemical characterization, we confirmed that the produced BC has a typical type *I*_α_ cellulose structure with good thermal stability and crystallinity. In addition, we also evaluated the adsorption capacity of BC produced in RFH medium for Cu(II) ions in aqueous solution. The results show that as the pH value increases, the adsorption efficiency of BC to Cu(II) increases significantly, which is mainly attributed to the increase in negative charges on the surface of BC, which is conducive to electrostatic attraction with positively charged Cu(II) ions. At pH 7.0, the adsorption efficiency of Cu(II) by BC reached 95.62%, showing its potential application value in treating textile industry wastewater. Overall, this study not only provides a new way for the high-value utilization of ramie textile waste, but also provides theoretical guidance and technical support for the industrial production and application of BC. Future research will focus on further optimizing the fermentation process, improving BC production efficiency, and exploring other potential applications in environmental remediation and biomedicine.

## Electronic supplementary material

Below is the link to the electronic supplementary material.

Supplementary Material 1

## Data Availability

The datasets generated during and/or analyzed during the current study are available from the corresponding author upon reasonable request. The sequencing data generated in this study have been deposited in the NCBI Sequence Read Archive (SRA) under BioProject accession number OR064102.1. The data are publicly available at: https://www.ncbi.nlm.nih.gov/search/all/?term=OR064102.1.
