# Supplementary material for: Bio-synthesis of bacterial cellulose from ramie textile waste for high-efficiency Cu(II) adsorption
Source: Sci Rep. 2025 May 28;15:18715. doi: 10.1038/s41598-025-02310-6 (PMC12120067; doi:10.1038/s41598-025-02310-6)
Supplement: Supplementary file 1 — Supplementary Material 1 [file 41598_2025_2310_MOESM1_ESM.docx]

**Supplementary Material**

**Bio-synthesis of Bacterial Cellulose from Ramie Textile Waste for High-Efficiency Cu(II) Adsorption**

**Table S1** Biochemical characterization of strain isolated from kombucha.

| Biochemical assay | Result |
| --- | --- |
| Gram staining | Gram-negative |
| Catalase | + |
| Oxidase | - |
| Oxidation of acetate | + |
| Oxidation of lactate | + |
| Oxidation of ethanol | + |
| Exercise | - |

Note: + indicates that the response is characterised as positive; - indicates that the response is characterised as negative.


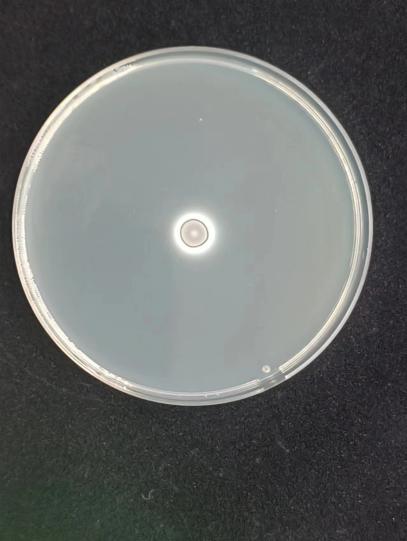

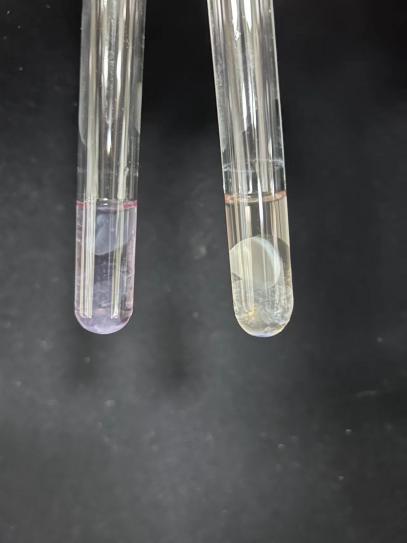


**(b)**

**(a)**


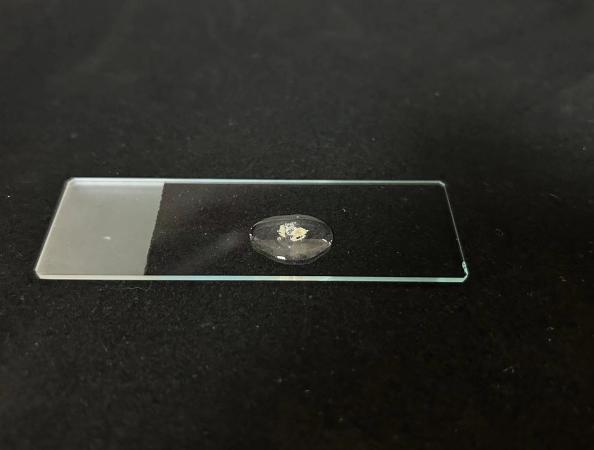


**(c)**

**Fig. S1** (a) Oxidation of ethanol test, (b) oxidation of acetate test, and (c) catalase test.


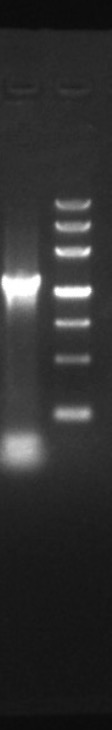


**Fig. S2** The original full-length imprinted image of electropherogram pattern.

**Single factor analysis**

Through the single factor experiment, it can be observed as follows (Fig. S1). (1) During the hydrolysis of ramie fibers, the hydrolysis efficiency of cellulose increased with the increase of enzyme dosage. When the usage of cellulase was 5%, the reducing sugar content in hydrolysate reached 26.736 g/L (Fig.S1). (2) Temperature, as one of the factors affecting the hydrolysis efficiency of ramie fibers, mainly affects the structure of enzyme protein through temperature. According to the trend diagram of ramie hydrolysis temperature, the reducing sugar produced by ramie hydrolysis reached the highest yield of 28.123 g/L at 40 ℃, indicating that the enzyme activity reached the highest. With the increase of temperature, cellulase activity, reducing sugar yield and hydrolysis efficiency of ramie decreased. (3) The effect of reaction time on the hydrolysis of ramie is mainly reflected in whether the hydrolysis is complete. When the reaction time was 60 h, the reducing sugar content in the hydrolysate reached 27.519 g/L. After 60 h, the content of lute fiber decreased, the rate of enzyme reaction decreased significantly, and the rate of increase of reducing sugar content decreased.


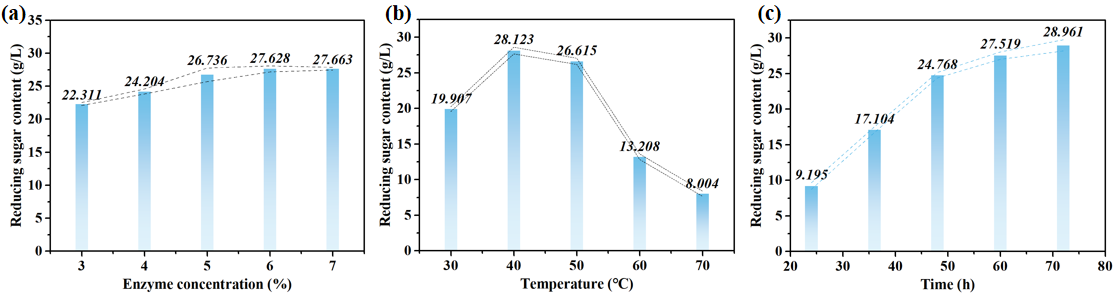


**Fig. S****3** Effect of enzyme concentration, temperature, and time on the yield of reducing sugars produced by ramie fibers hydrolysis

**Experimental design of response surface methodology**

Central composite design and response surface methodology of three factors at three levels were adopted, as shown in Table S2. Code values of three arguments, that is, temperaturethe (*x*_1_, ℃) and the enzyme concentrations (*x*_3_, %) and time (*x*_2_, h) were *X*_1_, *X*_2_ and *X*_3_, respectively. Each significant factor is coded at three levels, low (-1), medium (0), and high (+1). The following equation (Eq. S1) was used for coding the variables. The reducing sugar content was used as the response value to study the hydrolysis process.

*X*_i_=(*x*_i_-*x*_0_)/*∆*_i_  Eq. S1

where *X*i is the dimensionless value of an independent variable, *x*i is the independent variable, *x*0 is the value of *x*i at the equilibrium point, and Δi is the amount of step change.

**Table S2** Factors and levels for response surface optimization test

| Level | Independent Variables | | |
| --- | --- | --- | --- |
|  | Temperature*^A^*/°C | Enzyme Concentration*^B^*/% | Time*^C^*/h |
| -1 | 30 | 4 | 48 |
| 0 | 40 | 5 | 60 |
| 1 | 50 | 6 | 72 |

Factor code: *A*. *x*_1_; *B*. *x*_2_; *C*. *x*_3_.

**Modeling and the Test of Significance**

Experimental groups and results are given in Table S3. The runs from 1 to 12 were arrayed in the experiment to study the effects of variables on the preparation of BC and the runs from 13 to 17 were central experiments to assess experimental and systematic errors.

**Table S3** Box-Behnken experimental design and the corresponding response values

| Run | *x*_1_/℃ | *X*_1_ | *x*_2_/% | *X*_2_ | *x*_3_/h | *X*_3_ | *Y/*g/L |
| --- | --- | --- | --- | --- | --- | --- | --- |
| 1 | 30 | -1 | 4 | -1 | 60 | 0 | 20.25 |
| 2 | 50 | 1 | 4 | -1 | 60 | 0 | 19.265 |
| 3 | 30 | -1 | 6 | 1 | 60 | 0 | 23.683 |
| 4 | 50 | 1 | 6 | 1 | 60 | 0 | 24.735 |
| 5 | 30 | -1 | 5 | 0 | 48 | -1 | 15.912 |
| 6 | 50 | 1 | 5 | 0 | 48 | -1 | 16.326 |
| 7 | 30 | -1 | 5 | 0 | 72 | 1 | 24.269 |
| 8 | 50 | 1 | 5 | 0 | 72 | 1 | 25.914 |
| 9 | 40 | 0 | 4 | -1 | 48 | -1 | 20.641 |
| 10 | 40 | 0 | 6 | 1 | 48 | -1 | 24.052 |
| 11 | 40 | 0 | 4 | -1 | 72 | 1 | 29.571 |
| 12 | 40 | 0 | 4 | 1 | 72 | 1 | 29.552 |
| 13 | 40 | 0 | 5 | 0 | 60 | 0 | 30.963 |
| 14 | 40 | 0 | 5 | 0 | 60 | 0 | 30.803 |
| 15 | 40 | 0 | 5 | 0 | 60 | 0 | 29.528 |
| 16 | 40 | 0 | 5 | 0 | 60 | 0 | 29.176 |
| 17 | 40 | 0 | 5 | 0 | 60 | 0 | 28.946 |

With the help of Design-Expert 13 software, the experimental data were fitted with multivariable regression. Meanwhile, the fitting equation and significance of interrelated factors were analyzed. Then the fitting equation and the optimum process conditions of BC were obtained. Based on the multiple-regression analysis of experimental results, regression equation (Eq. S2) between factor and response value (reducing sugar content, *Y/*g/L) was obtained.

*Y*=29.88+0.2658*X*_1_+1.54*X*_2_+4.05*X*_3_+0.5092*X*_1_*X*_2_+0.3078*X*_1_*X*_3_-0.8575*X*_2_*X*_3_-6.62*X*_1_^2^-1.28*X*_2_^2^ -2.65*X*_3_^2^ Eq. S2

Regression coefficients and their significance based on response surface quadratic model as well as variance and notability analysis are shown in Table S3. As shown in Table S4, the *P*-value of regression model is lower than 0.0001 and the lack of fit is not significant (*P* = 0.229 > 0.05), indicating that this model equation is highly reliable and well-fitted to the actual situation. The determination coefficient (*R*^2^) of this model was 0.9767 and the adjusted determination coefficient (*R*^2^_Adj_) was 0.9466, suggesting that the model may explain 94.66% of the total variation in response and has a high degree of fitting. The value of CV% and adeq precision is respective to be 4.46% and 15.98, which further proves that the model equation is reliable.

*X*_2_, *X*_3_, *X*_1_^2^, and *X*_3_^2^ of model are highly significant, namely, the enzyme concentration, the time, and quadratic terms of temperature and time would statistically significantly affect the reducing sugar yield. These include the factors that influence largely the response value, i.e., the enzyme concentration, the time, and quadratic terms of temperature. The order of influent forces is *X*_1_^2^ ＞ *X*_3_ ＞ *X*_3_^2^ ＞ *X*_2_.

**Table S4** Variance analysis of regression model

| **Source** | **Quadratic sum** | Degree of freedom | **Mean Square** | ***F*-Value** | ***P*-Value** | Prob>F |
| --- | --- | --- | --- | --- | --- | --- |
| Model | 391.25 | 9 | 43.47 | 32.53 | <0.0001 | ** |
| *X*_1_-temperature | 0.565 | 1 | 0.565 | 0.4228 | 0.5363 |  |
| *X*_2_-Enzyme Concentration | 18.9 | 1 | 18.9 | 14.14 | 0.0071 | ** |
| *X*_3_-time | 131.02 | 1 | 131.02 | 98.05 | < 0.0001 | ** |
| *X*_1_*X*_2_ | 1.04 | 1 | 1.04 | 0.7763 | 0.4075 |  |
| *X*_1_*X*_3_ | 0.3788 | 1 | 0.3788 | 0.2835 | 0.6109 |  |
| *X*_2_*X*_3_ | 2.94 | 1 | 2.94 | 2.2 | 0.1815 |  |
| *X*_1_^2^ | 184.77 | 1 | 184.77 | 138.28 | < 0.0001 | ** |
| *X*_2_^2^ | 6.85 | 1 | 6.85 | 5.13 | 0.058 |  |
| *X*_3_^2^ | 29.65 | 1 | 29.65 | 22.19 | 0.0022 | ** |
| Residual | 9.35 | 7 | 1.34 |  |  |  |
| Lack of Fit | 5.84 | 3 | 1.95 | 2.21 | 0.229 |  |
| Pure Error | 3.52 | 4 | 0.8792 |  |  |  |
| Cor Total | 400.6 | 16 |  |  |  |  |

“*” indicates a significant effect on the results (p<0.05); “**” indicates a highly significant effect on the results (p<0.01)

**Table S5** Optimization of bacterial cellulose media

| **Type of medium** | **Incubation time(d)** | **Dry weight yield measured at full BC growth (g/L)** |
| --- | --- | --- |
| HS | 14 | 3.2 ± 0.12 |
| Ramie hydrolysate | 7 | 3.2±0.07 |
| Optimized medium of ramie hydrolysate | 7 | 7.2±0.14 |


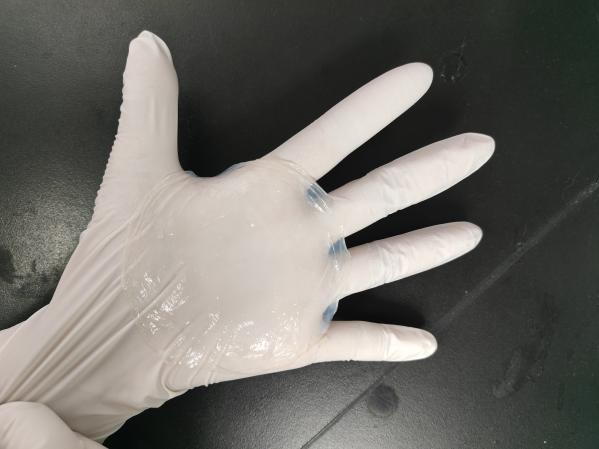

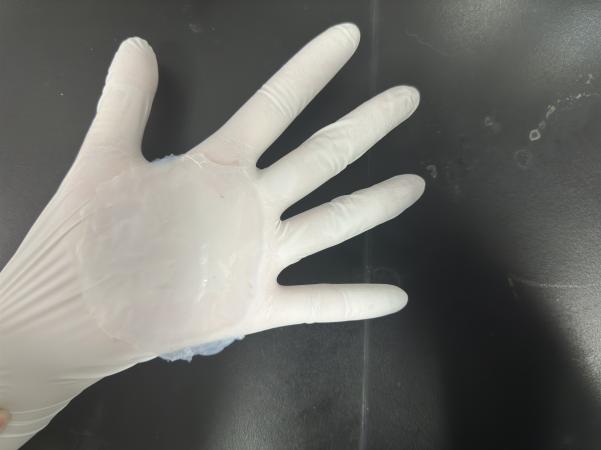


**(a)**

**(b)**

**Fig. S4** The pellicle formed by *Novacetimonas hansenii* HX1 strains at the surface of the RFH medium: (a) undried sample, (b) dried sample.


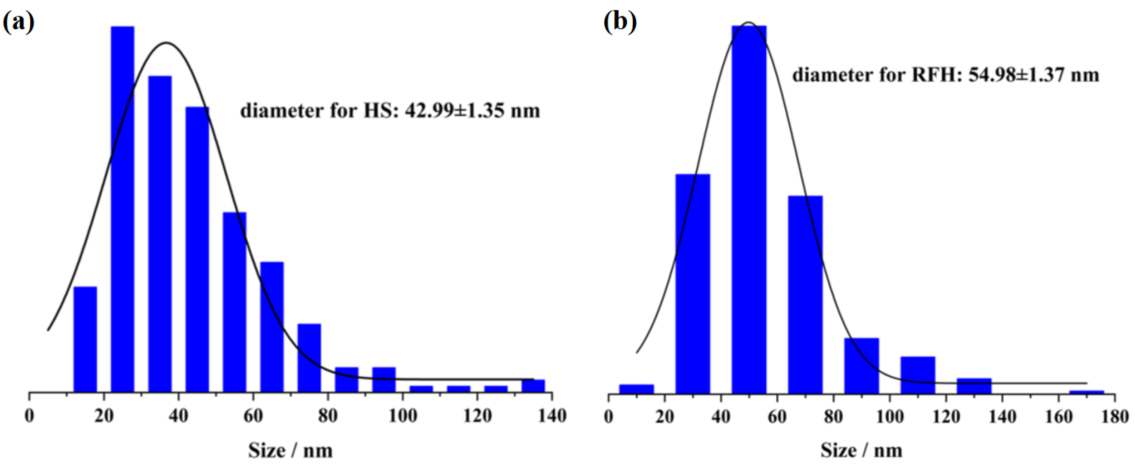


**Fig. S5** Fiber diameters of samples from HS (a) and RFH (b) media.

**Table S6** Full width at half maximum (FWHM), interplanar distances (d-spacing), crystallites size, and crystallinity degree of bacterial cellulose produced in RFH and HS medium.

***^A^* Ruan et al., 2016; Dubey et al., 2017.**

**References**

Ruan, C., Zhu, Y., Zhou, X. Effect of cellulose crystallinity on bacterial cellulose assembly. *Cellulose* 23, 3417-3427 (2016).

Dubey, S., Sharma, R.K., Agarwal, P. From rottengrapes to industrial exploitation: Komagataeibacter europaeus SGP37, a micro-factory for macroscale production of bacterial nanocellulose. *Int. J. Biol. Macromol.* 96, 52-60 (2017).

| Sample | 2*θ* | (h, k, l) | FWMH (°) | *d****^A^*** (nm) | *D* (nm) | Z value | Crystallinity (%) |
| --- | --- | --- | --- | --- | --- | --- | --- |
| RFH | 14.70 | (100) | 1.43 | 0.60 | 5.62 | +31.65 | 68.05 |
|  | 17.10 | (010) | 0.87 | 0.52 | 9.25 |  |  |
|  | 22.90 | (110) | 1.31 | 0.39 | 6.20 |  |  |
| HS | 14.50 | (100) | 1.33 | 0.61 | 6.05 | +10.57 | 82.09 |
|  | 16.80 | (010) | 0.89 | 0.53 | 9.04 |  |  |
|  | 22.70 | (110) | 1.26 | 0.39 | 6.44 |  |  |

**Table S7** Key temperatures from thermogravimetric analysis during the evaluated temperature range.

| Medium | *T*_onset_ (℃) | *T*_max_ (℃) |
| --- | --- | --- |
| RFH | 268 | 283 |
| HS | 306 | 326 |


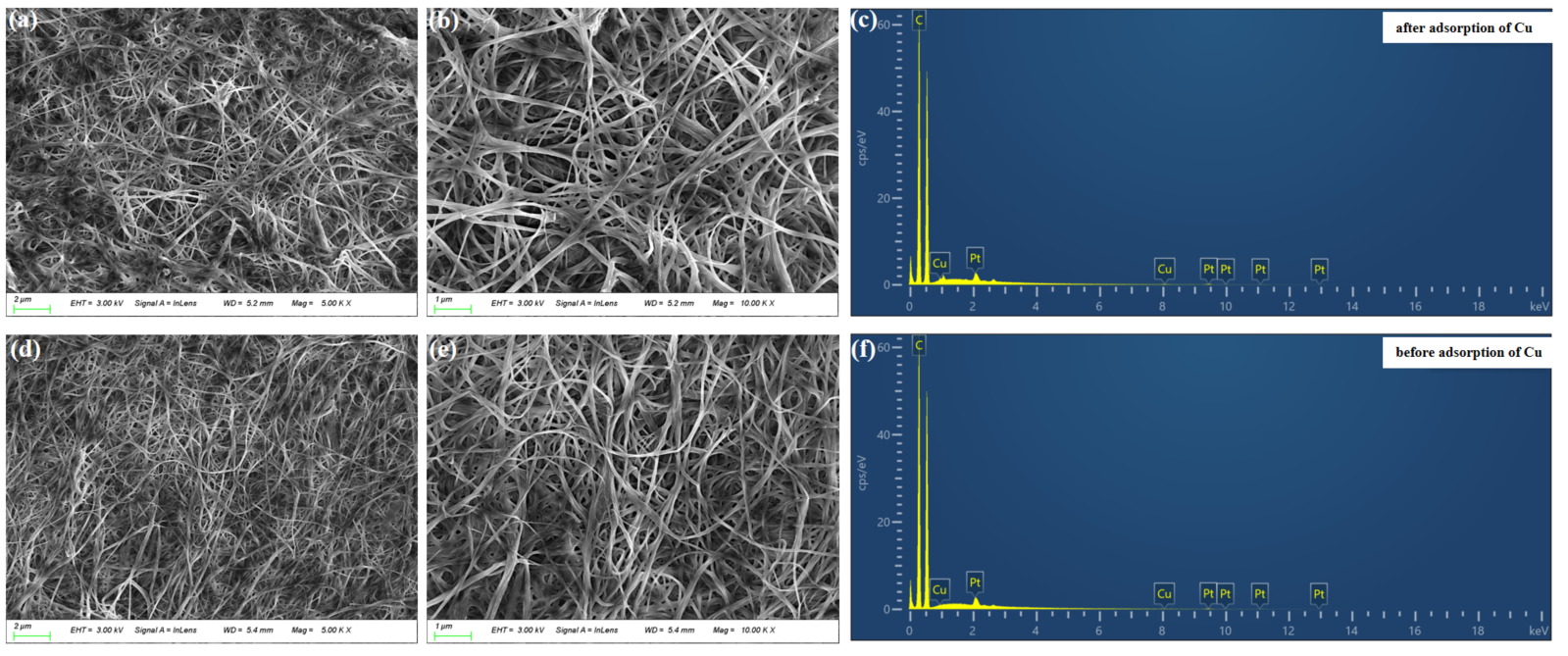


**Fig. S6** SEM images and EDX pattern of BC after (a-c) and before (d-f) adsorption of Cu
